# Evaluation of the relative weight (rw) of diagnosis-related groups (drg) in critically ill patients (cip) with severe sepsis (ss) according to the major diagnostic categories (mdc)

**DOI:** 10.1186/2197-425X-3-S1-A766

**Published:** 2015-10-01

**Authors:** J Ruiz Moreno, E González Marín, MJ Esteve Paños, R Corcuera Romero de la Devesa, MJ Riba Ribalta, S Godayol Arias, N Conesa Folch, F Baigorri González, A Artigas Raventós

**Affiliations:** Critical Care Department, QuirónSalud Hospital Universitario Sagrat Cor, Barcelona, Spain; Critical Care Department, Hospital de Sabadell & QuirónSalud Hospital Universitario Sagrat Cor, Sabadell, Spain

## Introduction

Apart from financial purposes (cost accounting), DRG as case - mix system are not used for obtaining competitive advantage. The variability of the RW of DRG related to SS has not been researched; it could be relevant from a socioeconomic perspective. In relation with SS, we hypothesize that the average RW of each MDC is different.

## Objectives

Analyze if the RW of the DRGs in CIPs with SS behaves differently depending on the MDC.

## Methods

- Type of Study: prospective, analytical, longitudinal, and observational
- Period: January 1-2011 / June 30-2014 (42 months)
- Setting : Medical/Surgical ICU
- Population: 2559 CIPs admitted consecutively to the ICU; sample: 484 CIPs with SS.
- Exclusión criteria: CIPs < 16 y., major burn CIPs, incomplete clinical documentation, and voluntary discharge.
- DRG AP-DRG 25.0 version (684 DRG are grouped into 25 Major Diagnostic Categories and 1 extra Category). Each DRG can be medical (M) or surgical (S).
- MDC: 1 (neurology), 2 (eye), 3 (ear, nose, mouth, throat), 4 (respiratory), 5 (circulatory), 6 (digestive, 7 (hepatobiliary & pancreas), 8 (musculoskeletal & connective), 9 (skin & breast), 10 (endocrine), 11 (urinary tract), 12 (male reproductive), 13 (female reproductive), 14 (pregnancy & childbirth), 15 (newborn), 16: (blood & immunological), 17 (mMyeloproliferative), 18 (infectious), 19 (mental), 20 (alcohol / drug), 21 (Injuries & poison), 22 (burns), 23 (factors influencing health status), 24 (HIV), 25 (PLT), 0 (PreMed, miscellany)
- Excluded MDC: 8 DRG with SS
- Depending on the focus of sepsis, SS related to MDC '0' (extra Category) are transferred to another MDC.
- Statistical analysis: ANOVA, ´F´ Snedecor. Scheffe's test post ANOVA to find out which pairs of MDC are significative.

## Results

See Tables 1 and 2.

**Table 1:** Results I.

MDC	8 DRG)	4 (114 DRG)	5 (30 DRG)	6 (152 DRG)	7 (94 DRG)	8 (14 DRG)	9 (8 DRG)	11 (22 DRG)	18 (33 DRG)
RW	8,46	11,06	6,19	9,93	4,50	5,11	4,03	5,16	3,76
MDC 1 (8 DRG)	NS	NS	NS	NS	NS	NS	NS	NS	NS
MDC 4 (114 DRG)	NS		0,01495	NS	0,0001	0,03020	0,04719	0,00989	0,00047
MDC 5 (30 DRG)	NS	0,01495S		NS	NS	NS	NS	NS	NS
MDC 6 (152 DRG)	NS	NS	NS		0,00015	NS	NS	0,03095	0,00165
MDC 7 (94 DRG)	NS	0,0001	NS	0,00015		NS	NS	NS	NS

**Table 2:** Results II.

MDC	1 (8 DRG)	4 (114 DRG)	5 (30 DRG)	6 (152 DRG)	7 (94 DRG)	8 (14 DRG)	9 (8 DRG)	11 (22 DRG)	18 /33 DRG)
RW	8,46	11,06	6,19	9,93	4,50	5,11	4,03	5,16	3,76
MDC 8 (14 DRG)	NS	0,03020	NS	NS	NS		NS	NS	NS
MDC 9 (8 DRG)	NS	0,04719	NS	NS	NS	NS		NS	NS
MDC 11 (22 DRG)	NS	0,00989	NS	0,03095	NS	NS	NS		NS
MDC 18 (33 DRG)	NS	0,00047	NS	0,00165	NS	NS	NS	NS

- Excluded MDC: 2, 3, 4, 12,13,14, 15, 16, 17, 19, 20, 21, 22, 23, 24 y 25
- Significative ANOVA (F Snedecor = 5,6633, P < 0,001):

## Conclusions

- The RW of MDC '4' and '6' is greater than the RW of the rest of the MDC.
- The RW of the rest of MDC are quite similar.
- MDC '4' and '6' differ, respectively, with 6 and 3 MDC.

